# Quantification of skeletal asymmetries in normal adolescents: cone-beam computed tomography analysis

**DOI:** 10.1186/s40510-014-0026-0

**Published:** 2014-04-01

**Authors:** Derek A Sanders, Taranpreet K Chandhoke, Flavio A Uribe, Paul H Rigali, Ravindra Nanda

**Affiliations:** 1 Private Practice, Miami, FL, USA; 2 Division of Orthodontics, Department of Craniofacial Sciences, University of Connecticut, School of Dental Medicine, 263 Farmington Avenue, Farmington CT 06030, USA; 3 Private Practice, Woodstock, VT, USA; 4 Department of Craniofacial Sciences, Alumni Endowed Chair, University of Connecticut, School of Dental Medicine, Farmington CT 06030, USA

## Abstract

**Background:**

The detection and quantification of skeletal asymmetries is a fundamental component to diagnosis and treatment planning in orthodontics. The purpose of this study was to identify and quantify the characteristics of facial and dental asymmetries in a normal, adolescent population using 3D imaging.

**Methods:**

Thirty consecutive Class I patients (mean age 14.32 years, SD 1.67) meeting the inclusion criteria were analyzed by three-dimensional cone-beam computed tomography (CBCT). Dental, maxillary, mandibular, and cranial base variables were measured with Dolphin 3D. CBCT analysis consisted of the localization of 34 anatomical landmarks. All reference points were digitized in 3D and analyzed using 67 skeletal and dental measurements. Student's *t* tests for paired samples were used with a significance level of *p* < 0.05.

**Results:**

Minor right-left discrepancies were noted in all planes. The most anterior point of the glenoid fossa and most condylar points were positioned more superior and lateral on the right side, compared to the left side. Porion was also located more superiorly on the right side relative to the left side. The posterior nasal spine was found to be located to the right of the midsagittal plane. Slight dental midline discrepancies were found, and the dental arch lengths were slightly longer on the left side compared to the right. The height of the ramus, in both 3D and 2D, and the inclination of the ramus were greater on the right than that on the left side.

**Conclusions:**

The findings of this study suggest minor asymmetries exist and are likely a common occurrence in the normal human craniofacial complex. Additionally, a natural compensatory mechanism may exist which controls the size and shape of specific tissues in order to maintain functional symmetry.

## Background

Dentofacial asymmetries can pose a significant challenge to orthodontic treatment and an accurate diagnosis is key to localize the asymmetry and determine the best treatment strategy. Severe forms of dentofacial deformities, such as those caused by syndromes like hemifacial microsomia, cleft palate, or hemimandibular hyperplasia, have been well described in the literature [1-3]. However, minor asymmetries are significantly more common in the general population but have not been studied as extensively. Asymmetries have been traditionally diagnosed through the analysis of postero-anterior (PA) X-rays, photographs and dental models [2,4,5]. Radiographs offer the benefit of measuring the skeletal component of an asymmetry, but 2D images, like PA cephalograms, are limited due to the presence of numerous artifacts, overlapping structures and magnification distortion [6,7]. The advent of three-dimensional imaging through cone-beam computed tomography (CBCT) offers the possibility of accurate localization and quantification of asymmetries without distortion. In patients with significant mandibular asymmetry, CBCT has been employed to quantify the degree of asymmetry with a high degree of accuracy compared to two-dimensional images [8-11]. No studies have yet been published with normative 3D values of asymmetry for patients with normal occlusions. The purpose of this study was to identify and quantify dentofacial asymmetries in a normal, adolescent population.

## Methods

### Subjects

**Figure 1:**
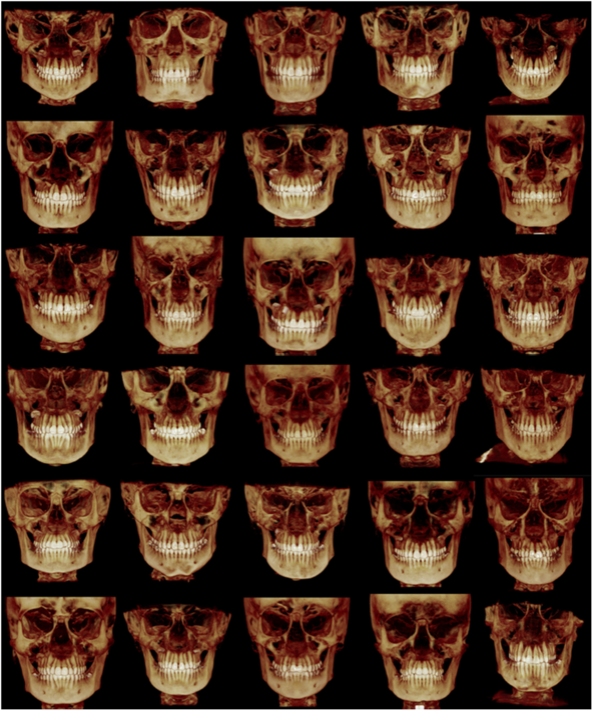
Volumetric images of each of the 30 Class I subjects included in the study.

This study was conducted in accordance with a protocol approved by the Institutional Review Board of the University of Connecticut (IRB #08-298-1). CBCT scans and records from two orthodontic offices (Dr. Rigali and Dr. Roy) that routinely use 3D imaging for comprehensive orthodontic diagnosis and treatment planning were reviewed. CBCT images, clinical examination records, dental models, and photographs were reviewed and subjects were selected based on the following inclusion criteria: (1) Class I molar relationship, (2) teeth in occlusion from second molar to second molar, (3) no malformed or missing teeth, (4) coincident dental and facial midlines, (5) no dental or skeletal asymmetries on clinical exam, (6) no previous orthodontic treatment, (7) no crossbites, (8) no history of facial trauma or medical conditions that may have altered growth, (9) symmetrical spacing or crowding up to 3 mm per arch, (10) age from 10 to 18. The final study sample was determined by two investigators (PHR and DS) and consisted of 30 consecutive patients (16 boys, 14 girls) with an average age of 14.32 years (SD 1.67 years). The sample included 28 Caucasians, 1 Asian, and 1 African-American. Figure 1 shows volumetric frontal views for each patient included in this study.

### CBCT acquisition

Full-head CBCT scans (i-CAT Classic, Imaging Sciences International, Hatfield, PA, USA) were acquired with patients in maximum intercuspation. A 16.0 cm (diameter) × 13.0 to 22.0 cm (height) field of view at a resolution of 0.4 mm voxels was used for each volumetric data set with an acquisition setting of 120 kVp and 5 mA based on the manufacturer's specifications. The scan times ranged from 20 (13.0 cm) to 40 s (22.0 cm) depending on the vertical height of the field of view selected by the clinician based on patient factors like the skull size and height. Reconstructed data (by Xoran i-CAT software, version 2.1.22) was exported as a 12-bit-depth digital imaging and communications in medicine (DICOM) file. Final analysis of the DICOM data was completed using Dolphin 3D (version 10.5, Dolphin Imaging, Chatsworth, CA, USA).

### CBCT orientation and landmark identification

**Figure 2:**
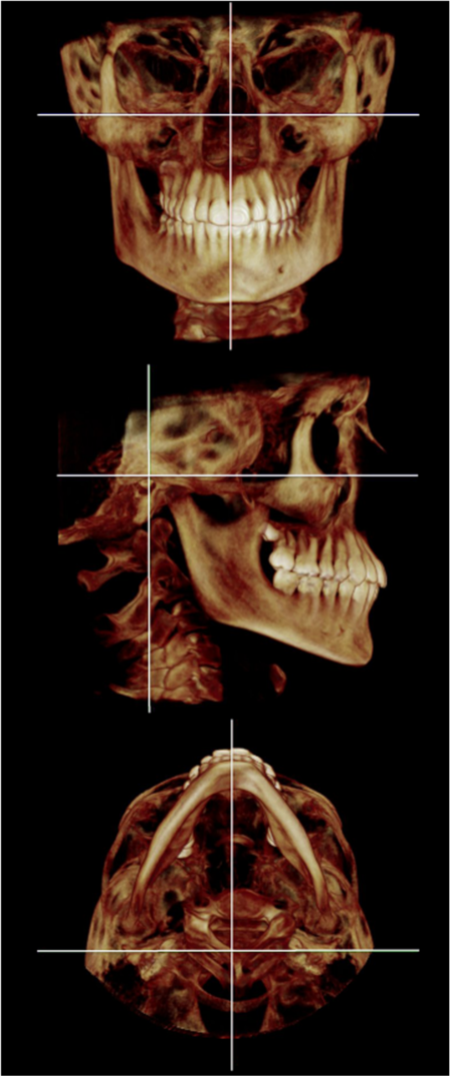
Volumetric renderings of the reference planes used in the study.

Orientation of each data set was completed in three planes of space (*x*, *y*, and *z*) using volumetric rendering and multiple planar views within Dolphin 3D (Figure 2). First, the sagittal plane (*x*) was constructed bisecting paired midfacial anatomic structures (i.e., orbits, frontal process of the maxilla, and frontozygomatic suture). The axial plane (*y*) was then constructed as a line connecting the most superior point of the external acoustic meatus (porion) with the most inferior point of the infraorbital rim (orbitale) on the right and left sides, which would correspond similarly to the Frankfort horizontal plane in 2D. Finally, the coronal plane (*z*) was constructed using the transporionic line and fixed perpendicular to the axial and sagittal planes. Using modified orthodontic cephalometric landmarks, 34 anatomic landmarks were defined and digitized (Table 1) [12]. Coordinate data for each defined landmark was exported to an Excel database (Excel 2007, Microsoft, Redmond, WA, USA) and orthogonal, linear, and angular measurements were calculated.

**Table 1:** Study landmarks and reference planes

**Landmarks**			**Definition**	**Sagittal slice**	**Axial slice**	**Coronal slice**
Dental	Mx1	Maxillary central incisors	Incisal contact point of the maxillary central incisors	Inferior-most point	Contact point	Incisal embrasure
	Md1	Mandibular central incisors	Incisal contact point of the mandibular central incisors	Inferior-most point	Contact point	Incisal embrasure
	Mx3_R_ and Mx3_L_	Maxillary canine	Cusp tip of the maxillary canine	Inferior-most point	First point that appears	Inferior-most point
	Md3_R_ and Mx3_L_	Mandibular canine	Cusp tip of the mandibular canine	Superior-most point	First point that appears	Superior-most point
	Mx6_R_ and Mx6_L_	Maxillary first molar	Mesiobuccal cusp tip of the maxillary first molar	Inferior-most point	First point that appears	Middle-inferior-most point
	Md6_R_ and Mx6_L_	Mandibular first molar	Buccal groove of the mandibular first molar	Inferior-most point between the buccal cusps	First point that appears	Superior-most point in line with the buccal cusps
Midface	ANS	Anterior nasal spine	The most anterior midpoint of the anterior nasal spine of the maxilla	Lateral-most point	Superior-most point	First point that appears
	PNS	Posterior nasal spine	The most posterior midpoint of the posterior nasal spine of the palatine bone	Lateral-most point	Inferior-most point	First point that appears
	Or_R_ and Or_L_	Orbitale	The most inferior point on the infraorbital rim of the maxilla	Inferior-most point of the infraorbital rim	Anterior-most point	Inferior-most point of the infraorbital rim
	FZS_R_ and FZS_L_	Frontozygomatic suture	The most superior and medial point on the frontozygomatic suture	Superior-most point	Anterior-most point	Medial-superior most point
	FPM_R_ and FPM_L_	Frontal process of maxilla	Intersection of the frontal process of the maxilla, frontal bone and lacrimal bone	Superior-most point	Anterior-most point	Medial-superior most point
Mandible	Me	Menton	The most inferior midpoint of the chin on the outline of the mandibular symphysis	Inferior-most point	First point that appears	Middle-most point along the inferior border
	Pg	Pogonion	The most anterior midpoint of the chin on the outline of the mandibular symphysis	Inferior-most point	Superior-most point	First point that appears
	Go_R_ and Go_L_	Gonion	The midpoint on the angle of the mandible, half way between the corpus and ramus	Middle-most point along the angle of the mandible	Middle-inferior-most point	Middle-inferior-most point
	CdS_R_ and CdS_L_	Condyle superior	The most superior point of the condylar head	Superior-most point	First point that appears	Superior-most point
	CdL_R_ and CdL_L_	Condyle lateral pole	The most lateral point of the condylar head	First point that appears	Lateral-most point of the widest section of the condyle	Lateral-most point
	CdM_R_ and CdM_L_	Condyle medial pole	The most medial point of the condylar head	First point that appears	Medial-most point of the widest section of the condyle	Medial-most point
	CdA_R_ and CdA_L_	Condyle anterior	The most anterior point of the condylar head	Anterior-most point	Middle-most point on the anterior surface of the widest section of the condyle	Medial-most point
	CdP_R_ and CdA_L_	Condyle posterior	The most posterior point of the condylar head	Posterior-most point	Middle-most point on the posterior surface of the widest section of the condyle	N/A
Temporal bone	GlS_R_ and GlS_L_	Glenoid fossa superior	The most superior point of the glenoid fossa of the temporal bone	Superior-most point	First point that appears	Superior-most point
	GlA_R_ and GlA_L_	Glenoid fossa Anterior	The most inferior point of the articular eminence of the temporal bone	Inferior-most point on the articular eminence	Anterior-most point	Superior-most point
	Po_R_ and Po_L_	Porion	The most superior point of the external acoustic meatus	Superior-most point of the external acoustic meatus	First point that appears	Superior-most point of the external acoustic meatus
Reference planes						
Axial plane			A plane that connects the most superior point of the external acoustic meatus with the most inferior point of the infraorbital rim on the right and left sides			
Sagittal plane			A plane constructed using paired midfacial anatomic structures (eg., the orbits, frontal process of the maxilla, frontozygomatic suture)			
Coronal plane			A plane constructed from the transporionic line			

### Measurements

Orthogonal measurements were defined using sagittal, axial, and coronal planes and measured as perpendicular millimeter distances from the specific 3D landmark to each of the reference planes. Dental and dentoalveolar asymmetry, mandibular morphologic asymmetry, and condylar morphologic asymmetry were expressed as linear and angular measurements in millimeters and degrees, respectively (Table 2).

**Table 2:** Definition of measurements

	**Variable**	**Definition**
Dental	A-P molar differential (mm)	The difference of Mx6 (*z*) and Md6 (*z*)
	Midline differential (mm)	The absolute difference of Mx1 (*x*) and Md1 (*x*)
	Overbite (mm)	Mx1 (*y*) minus Md1 (*y*)
	Overjet (mm)	Mx1 (*z*) minus Md1 (*z*)
	Maxillary arch length (mm)	Distance (*x*, *y*, *z*) between Mx6 and Mx1
	Mandibular arch length (mm)	Distance (*x*, *y*, *z*) between Md6 and Mx1
Midface	Palatal plane to the sagittal plane (^o^)	Angulation (*x*, *z*) between ANS-PNS and the sagittal plane
Mandibular	Mandibular length in 3D (mm)	Distance (*x*, *y*, *z*) between CdS and Pg
	Mandibular length in 2D (mm)	Distance (*y*, *z*) between CdS and Pg
	Ramus height in 3D (mm)	Distance (*x*, *y*, *z*) between CdS and Go
	Ramus height in 2D (mm)	Distance (*y*, *z*) between CdS and Go
	Corpus length in 3D (mm)	Distance (*x*, *y*, *z*) between Go and Pg
	Corpus length in 2D (mm)	Distance (*y*, *z*) between Go and Pg
	Gonial angle (^o^)	Angulation (*y*, *z*) between CdS, Go, and Pg
	Mandibular plane angle (^o^)	Angulation (*y*, *z*) between Go-Pg and the Frankfort horizontal
	Ramus inclination to the sagittal plane (^o^)	Angulation (*x*, *y*) between CdL-Go and the sagittal plane
	Dental and chin inclination (^o^)	Angulation (*x*, *y*) between Md1-Me and the sagittal plane
Condylar	Mediolateral diameter of condylar head in 3D (mm)	Distance (*x*, *y*, *z*) between CdL and CdM
	Mediolateral diameter of condylar head in 2D (mm)	Distance (*x*, *y*) between CdL and CdM
	Anteroposterior diameter of condylar head in 3D (mm)	Distance (*x*, *y*, *z*) between CdA and CdP
	Anteroposterior diameter of condylar head in 2D (mm)	Distance (*y*, *z*) between CdA and CdP
	Superior joint space in 3D (mm)	Distance (*x*, *y*, *z*) between CdS and GlS
	Superior joint space in 2D (mm)	Distance (*x*, *y*) between CdS and GlS
	Condylar head inclination to the coronal plane (^o^)	Angulation (*x*, *z*) between CdL-CdM and the coronal plane
	Condylar head inclination to the axial plane (^o^)	Angulation (*x*, *y*) between CdL-CdM and the axial plane

### Statistical analysis

One investigator (DS) completed all measurements and intrarater reliability was assessed using the intraclass correlation coefficient (ICC) on a random sample of 10 patients, with each variable measured at two time points, 2 weeks apart. Once intrarater reliability was established, right-left and absolute measurements were analyzed using Student's *t* tests for paired samples in the SPSS software program (version 14.0.1, SPSS, Chicago, IL, USA) with a significance of *p* < 0.05.

## Results

Intrarater reliability was measured, with high ICC values for each variable in all three reference planes as reported previously [12]. Measurements are presented as mean values ± the standard deviation for each point described. For orthogonal differences relative to the axial plane (Table 3), glenoid fossa anterior (GlA) was found to be placed 0.47 ± 1.08 mm superiorly on the right side relative to the left (*p* < 0.05). In addition, porion (Po) was also found to be positioned 0.37 ± 0.88 mm superiorly on the right side (*p* < 0.05). Measurements to the coronal plane were all found to be non-significant (Table 4).

**Table 3:** Orthogonal left-right differences from axial plane

	**Variable**	**Mean**	**SD**	**95%confidence interval**		***p*** **value**
				**Lower**	**Upper**	
Dental	Mx3 (*y*) to the axial plane (mm)	−0.11	0.96	−0.46	0.25	0.55
	Md3 (*y*) to the axial plane (mm)	0.00	0.62	−0.24	0.23	0.98
	Mx6 (*y*) to the axial plane (mm)	0.12	0.90	−0.22	0.46	0.47
	Md6 (*y*) to the axial plane (mm)	−0.01	0.88	−0.34	0.32	0.95
Midface	Or (*y*) to the axial plane (mm)	−0.09	0.75	−0.37	0.19	0.52
	Go (*y*) to the axial plane (mm)	0.5	1.43	−0.03	1.03	0.07
Mandible	CdS (y) to the axial plane (mm)	−0.22	0.93	−0.56	0.13	0.21
	CdL (*y*) to the axial plane (mm)	0.03	1.12	−0.39	0.45	0.9
	CdM (*y*) to the axial plane (mm)	−0.14	1.07	−0.54	0.26	0.49
	CdA (*y*) to the axial plane (mm)	−0.02	1.01	−0.4	0.36	0.91
	CdP (*y*) to the axial plane (mm)	−0.05	1.02	−0.43	0.33	0.79
Temporal bone	GlS (*y*) to the axial plane (mm)	−0.12	0.76	−0.4	0.17	0.41
	GlA (*y*) to the axial plane (mm)	−0.47	1.08	−0.87	−0.06	0.025*
	Po (*y*) to the axial plane (mm)	−0.37	0.88	−0.7	−0.04	0.03*

*p < 0.05.

**Table 4:** Orthogonal left-right differences from coronal plane

	**Variable**	**Mean**	**SD**	**95%confidence interval**		***p*** **value**
				**Lower**	**Upper**	
Dental	Mx3 (*z*) to the coronal plane (mm)	0.00	0.94	−0.35	0.35	0.94
	Md3 (*z*) to the coronal plane (mm)	−0.01	0.77	−0.30	0.27	0.93
	Mx6 (*z*) to the coronal plane (mm)	0.03	1.00	−0.34	0.41	0.86
	Md6 (*z*) to the coronal plane (mm)	0.00	0.98	−0.37	0.36	0.99
Midface	Or (*z*) to the coronal plane (mm)	−0.02	1.48	−0.58	0.53	0.93
	Go (*z*) to the coronal plane (mm)	0.06	1.66	−0.56	0.68	0.84
Mandible	CdS (*z*) to the coronal plane (mm)	0.10	2.35	−0.78	0.97	0.82
	CdL (*z*) to the coronal plane (mm)	0.02	2.12	−0.77	0.81	0.96
	CdM (*z*) to the coronal plane (mm)	0.13	2.33	−0.74	1.00	0.77
	CdA (*z*) to the coronal plane (mm)	−0.54	2.11	−1.32	0.25	0.17
	CdP (*z*) to the coronal plane (mm)	−0.07	2.01	−0.82	0.68	0.84
Temporal bone	GlS (*z*) to the coronal plane (mm)	−0.08	2.39	−0.97	0.81	0.86
	GlA (*z*) to the coronal plane (mm)	−0.82	2.24	−1.65	0.02	0.06
	Po (*z*) to the coronal plane (mm)	0.19	1.72	−0.45	0.83	0.54

Significant differences were seen within the condylar and glenoid fossa points in the measurements to the sagittal plane (Table 5). Among the condylar measurements, the medial pole (CdM), anterior pole (CdA), and posterior pole (CdP) were all found to be more laterally placed on the right side compared to the left, with a discrepancy of 0.62 ± 1.28 mm for CdM (*p* < 0.05), 1.34 ± 1.47 mm for CdA (*p* < 0.001), and 0.9 ± 1.51 mm for CdP (*p* < 0.01). The GlA was also more laterally placed, by 1.73 ± 1.52 mm, on the right side relative to the left side (*p* < 0.001).

**Table 5:** Orthogonal left-right differences from sagittal plane

	**Variable**	**Mean**	**SD**	**95%confidence interval**		***p*** **value**
				**Lower**	**Upper**	
Dental	Mx3 (*x*) to the saggital plane (mm)	−0.02	1.11	−0.43	0.40	0.94
	Md3 (*x*) to the saggital plane (mm)	−0.25	1.46	−0.79	0.30	0.36
	Mx6 (*x*) to the saggital plane (mm)	0.25	1.13	−0.17	0.67	0.24
	Md6 (*x*) to the saggital plane (mm)	0.29	1.07	−0.11	0.69	0.16
Midface	Or (*x*) to the saggital plane (mm)	−0.05	1.70	−0.69	0.58	0.87
	Go (*x*) to the saggital plane (mm)	0.48	1.89	−0.22	1.19	0.17
Mandible	CdS (*x*) to the saggital plane (mm)	−0.19	1.81	−0.86	0.49	0.57
	CdL (*x*) to the saggital plane (mm)	−0.55	1.56	−1.14	0.03	0.06
	CdM (x) to the saggital plane (mm)	−0.62	1.28	−1.10	−0.14	0.013*
	CdA (*x*) to the saggital plane (mm)	−1.34	1.47	−1.89	−0.80	0.0001***
	CdP (*x*) to the saggital plane (mm)	−0.90	1.51	−1.47	−0.34	0.003**
Temporal bone	GlS (*x*) to the saggital plane (mm)	0.24	1.54	−0.33	0.81	0.4
	GlA (*x*) to the saggital plane (mm)	−1.73	1.52	−2.30	−1.16	0.0001***
	Po (*x*) to the saggital plane (mm)	−0.35	1.66	−0.97	0.27	0.25

*p < 0.05, **p < 0.01, ***p < 0.001.

Midline structures were also measured to the sagittal plane (Table 6). Posterior nasal spine (PNS) was found to be 0.22 ± 0.54 mm to the right of the sagittal plane. No significance was noted with other midline structures.

**Table 6:** Orthogonal measurements of midline structures to sagittal plane

	**Variable**	**Mean**	**SD**	**95%confidence interval**		***p*** **value**
				**Lower**	**Upper**	
Dental	Mx1 (*x*) to the saggital plane (mm)	−0.11	0.51	−0.3	0.09	0.26
	Md1 (*x*) to the saggital plane (mm)	−0.11	0.56	−0.32	0.1	0.29
Midface	ANS (*x*) to the saggital plane (mm)	−0.16	0.48	−0.34	0.02	0.08
	PNS (*x*) to the saggital plane (mm)	−0.22	0.54	−0.43	−0.02	0.032*
Mandible	Me (*x*) to the saggital plane (mm)	0.11	0.68	−0.14	0.36	0.38
	Pg (*x*) to the saggital plane (mm)	0.2	0.67	−0.05	0.45	0.11

*p < 0.05.

For absolute measurements, distances between two landmarks on each side were measured and the difference compared between the right and left sides (Table 7). Dental midline differential (MLD), defined as the absolute difference between the incisal embrasure of the maxillary central incisors (Mx1) and the incisal embrasure of the mandibular incisors (Md1) from the sagittal plane, showed a significant discrepancy of 0.15 ± 0.20 mm (*p* < 0.0001). Maxillary (MXA) and mandibular (MDA) arch lengths were found to be longer on the left side *(p* < 0.05*)*, with the MXA left-right differential being 0.28 ± 0.66 mm and MDA equivalent to 0.33 ± 0.68 mm. Ramus height, defined as the distance between the most superior point of the condylar head (CdS) and gonion (Go), was found to be significantly greater on the right side (*p* < 0.05) using both 3D (RH3) and 2D (RH2) calculations. Absolute measurement differences between the left and right sides were 0.81 ± 1.65 for RH3 and 0.75 ± 1.60 mm for RH2. From the frontal aspect, ramus inclination (RI), measured as the angulation between most lateral point on the condylar head (CdL) and Go to the sagittal plane was significantly more obtuse on the right side by 1.22° ± 2.92° (*p* < 0.05).

**Table 7:** Absolute measurements of left-right differences

**Variable**	**Mean**	**SD**	**95%confidence interval**		***p*** **value**
			**Lower**	**Upper**	
A-P molar differential (mm)	0.04	0.28	−0.07	0.14	0.484
Midline differential (mm)	0.15	0.20	0.07	0.23	0.000****
Maxillary arch length (mm)	0.28	0.66	0.04	0.53	0.025*
Mandibular arch length (mm)	0.33	0.68	0.08	0.58	0.012*
Mandibular length in 3D (mm)	−0.43	1.53	−1.00	0.14	0.132
Manibular length in 2D (mm)	−0.21	1.53	−0.78	0.36	0.452
Ramus height in 3D (mm)	−0.81	1.65	−1.42	−0.19	0.012*
Ramus height in 2D (mm)	−0.75	1.60	−1.34	−0.15	0.016*
Corpus length in 3D (mm)	0.09	1.27	−0.38	0.57	0.690
Corpus length in 2D (mm)	0.08	1.67	−0.55	0.70	0.801
Gonial angle (°)	0.48	2.48	−0.44	1.40	0.297
Mandibular plane angle (°)	0.57	1.66	−0.06	1.19	0.073
Ramus inclination to the saggital plane (°)	−1.22	2.92	−2.32	−0.13	0.029*
Mediolateral diameter of the condylar head in 3D (mm)	0.06	1.22	−0.39	0.52	0.781
Mediolateral diameter of the condylar head in 2D (mm)	0.07	1.22	−0.38	0.52	0.752
Anteroposterior diameter of the condylar head in 3D (mm)	−0.24	0.85	−0.55	0.08	0.141
Anteroposterior diameter of the condylar head in 2D (mm)	−0.23	0.86	−0.55	0.09	0.149
Superior joint space in 3D (mm)	−0.03	0.78	−0.32	0.26	0.820
Superior joint space in 2D (mm)	0.08	0.63	−0.16	0.31	0.505

*p < 0.05, ****p < 0.0001.

## Discussion

The primary objective of this study was to measure the degree of asymmetry in patients who were otherwise defined as symmetric by clinical and radiographic examinations. The findings of this study show that skeletal asymmetries, while minor, may exist in otherwise clinically symmetric patients, confirming previous studies using 2D imaging and photography [6,13,14]. With the advent of CBCT technology, these small discrepancies can be localized to distinct sites. This data alludes to the possibility that during even the most tightly coupled processes, minor right-left discrepancies may be reflections of minute skeletal compensations that occur as growth is directed.

The asymmetries from this sample of patients showed either a mild right-side predominance or a mild left-side deficiency in the auriculo-temporal and condylar regions. The glenoid fossa was more laterally displaced on the right side and, along with the porion, was more superior in relation to the axial plane compared to the left side. Additionally, the condylar points were all more laterally displaced and the ramus heights were longer on the right side in both 3D and 2D. The ramus inclination was also more obtuse on the right side compared to the left, and the PNS point was shown to lie to the right of the midsagittal plane. The only measurements increased on the left side were maxillary and mandibular arch lengths. The slight right-side predominance seen in this study is consistent with a number of studies which have also shown an increased tendency for right side laterality, albeit with varying degrees of significance [6,8,13,15,16].

While the MLD showed a minor discrepancy in the maxillary and mandibular midlines, it should be noted that the lateral positions of menton (Me) and pogonion (Pg) relative to the midsagittal plane were not significant. This suggests that other compensations are likely to have occurred which adapted to the increased laterality and different vertical positions of the glenoid fossa and condylar points.

The intricate biological processes that direct growth and result in skeletal asymmetries have yet to be fully dissected. In his classic work, Woo proposed that minor asymmetries were due to skeletal compensations for asymmetric growth of the brain [17] and Burke theorized that right-left discrepancies were adaptive changes in response to asymmetric muscular and masticatory function [18]. At the molecular level, a number of genes and factors have been associated with asymmetric growth, identified in models of severe asymmetry associated with such syndromes as hemifacial microsomia, craniosynostosis and craniofacial clefts [19]. While genes, like Msx1, Goosecoid, TWIST, and TCOF1 have been linked to a number of craniofacial syndromes, no single gene has been identified as the sole cause of the skeletal abnormalities [20-23]. It is possible that a number of genes found within similar chromosomal loci are involved in these syndromes due to compromise during the embryonic period. Protein factors such as IGF-I, BMP-2 and TGF-β1 have been shown to be highly expressed in specific regions of the condylar cartilage in patients with condylar hyperplasia, a primary cause of skeletal asymmetry [24]. Proper functioning and regulation of these genes and factors is likely to promote normal symmetric growth. It is possible that minor, localized gene expression differences or altered growth factor activity may result in the small skeletal discrepancies noted in the normal patients in this study. Further research is needed in this area to better understand the complex biologic pathways involved in normal growth and to determine which molecular alterations lead to increased degrees of asymmetry in patients.

This study was the first of its kind to utilize CBCT to evaluate asymmetries in a normal, Class I, adolescent population. Previous studies in Class III patients have shown a tendency towards significant asymmetry, specifically with deviation of the lateral position of menton to the mid-sagittal plane when measured on PA cephalograms [25]. A study by Sievers and colleagues using CBCT imaging showed minor asymmetries in Class I and Class II patients using an asymmetry index (AI), a mathematical derivation which combined measurements from each landmark to the *x*-, *y*-, and *z*-planes [26]. The degree of AI was similar between both groups, which were characterized according to ANB angle. A recent study of 10 human dry skulls defined as exhibiting apparent symmetry were also shown to have minor asymmetries by CBCT analysis, with measurements highly accurate when compared to the gold standard, physical measurements [8]. In the same study, PA cephalograms where shown to have inconsistencies in measurements, likely due to the difficulty in visualizing key structures in the 2D view.

In addition to the skeletal component, the soft tissue plays an important role in asymmetry and requires further study. A number of studies have used standardized photographs and stereophotogrammetry to assess soft tissue asymmetry [16,18,27]. A hierarchy for anatomic landmarks has been previously established for constructing the soft tissue midline, with the natural dental midline and the tip of philtrum being most reliable for measuring soft tissue asymmetries in 2D [28]. The tip of the nose and soft tissue nasion have been shown to be less reliable in photographic measurements. The accuracy of soft tissue measurements with CBCT enables 1:1 accuracy and the ability to more clearly define soft tissue landmarks through superimposition on cranial structures [29]. Soft tissue asymmetry has yet to be measured in class I patients with CBCT, and this would help further understand the soft-tissue compensations that overcome the minor skeletal discrepancies we observed in this study.

There were a number of limitations of this study that should be considered. The right-left discrepancies detected in this sample of patients were very minor, with most less than 1.0 mm. For this reason, there is potential for some of these differences to be attributed to methodological error. Firstly, correct orientation of the CBCT in all three planes could have been a source of error. The method of sequential reference plane selection, particularly the use of paired midfacial structures for the midsagittal plane, was based on previously published studies [30,31]. While the sagittal plane was selected first, images also had to be corrected for *roll* to ensure the paired midfacial structures were correctly aligned vertically. The axial plane was then aligned for *pitch* and perpendicular to the sagittal plane followed by the coronal plane which was perpendicular to the sagittal and coronal planes and the best fit to the transporionic line. The ‘best fit’ of the three planes was determined by a single examiner at one timepoint. While the ICC for intrarater measurements was high, any minor inaccuracy in orientation of the CBCT at the initial stage could have resulted in some of the measured right-left differences seen in this study.

Landmark identification was another potential source of error in this study. Previous studies have shown the mediolateral dimension to be the least reproducible of the three dimensions with regards to landmark selection [26,32]. Particular anatomic landmarks pose challenges due to variation in morphology and convexity, such as condylion, gonion, and porion and there can be a greater degree of variability in the selection as shown by intra- and interrater reliability measures [33,34]. It is possible that utilizing a volumetric rendering view in conjunction with multiplanar views, as was used in this study, may minimize the degree of landmark selection error [35]. A number of the landmarks showing significant differences in this study were associated with these morphological challenges and thus could be skewed to show a right-left predominance or lack of predominance based on landmark selection.

The findings of this study reveal that very minor asymmetries may exist in otherwise symmetric patients, confirming previous studies that suggest that these discrepancies are normal and may be a reflection of a homeostatic mechanism during growth and bone remodeling.

## Conclusions

Minor asymmetries are present in all planes by CBCT analysis in a normal, adolescent population. The craniofacial complex may have a natural compensatory mechanism to control the size and shape of specific tissues in order to maintain functional symmetry and homeostasis. Further studies are needed to better understand the soft tissue component to asymmetries in this population of subjects.

## Competing interests

The authors declare that they have no competing interests.

## Authors’ contributions

DS carried out the study, collected the CBCT images, and made the measurements. TC drafted the manuscript and was involved in data analysis and final submission. FU was a primary advisor on the study and was involved in the design of the study and data analysis, as well as manuscript preparation. PR provided the CBCT records and was an advisor, meeting routinely on design and data collection. RN was an advisor on the study and involved in the manuscript preparation as well as guidance throughout the study on design and implementation. All authors read and approved the final manuscript.
